# Supplementary material for: Dietary Interventions in Ulcerative Colitis: A Systematic Review of the Evidence with Meta-Analysis
Source: Nutrients. 2023 Sep 28;15(19):4194. doi: 10.3390/nu15194194 (PMC10574654; doi:10.3390/nu15194194)
Supplement: Supplementary file 1 [file nutrients-15-04194-s001.zip › nutrients-2622186-supplementary.pdf]

	Random sequence generation (selection bias)	Allocation concealment (selection bias)	Blinding of participants and personnel (performance bias)	Blinding of outcome assessment (detection bias)	Incomplete outcome data (attrition bias)	Selective reporting (reporting bias)	Other bias
Bhattacharyya (2017)	−	−	+	?	?	−	+
Bodini (2019)	+	+	−	−	+	+	?
Candy (1995)	?	?	−	+	+	+	+
Fritsch (2021)	+	−	−	?	−	+	+
González–Huix (1993)	?	−	−	+	+	+	?
Ishikawa (2003)	+	+	−	−	+	+	?
Kato (2004)	+	+	−	−	+	+	?
Keshteli (2022)	?	?	+	?	+	+	?
Kyaw (2014)	+	+	−	−	+	+	?
Miyaguchi (2023)	+	+	−	−	+	+	?
Sarbagili–Shabat (2022)	+	+	−	−	+	+	?
Strisciuglio (2013)	+	?	−	+	+	+	+
Wright (1965)	?	?	−	?	−	−	?
Wright (1966)	?	?	−	?	−	−	?

Figure S1. Risk of bias summary

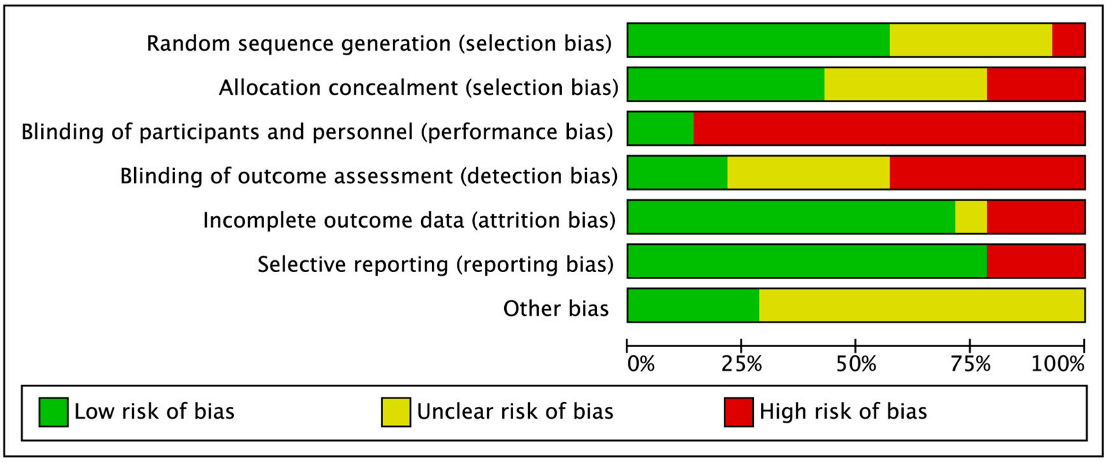

Figure S2. Risk of bias graph

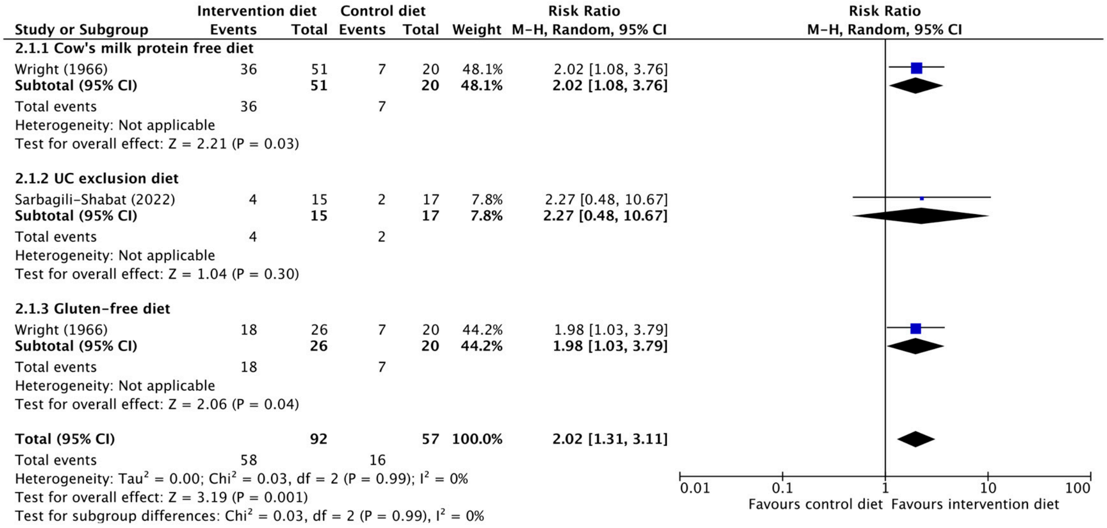

Figure S3. Induction of endoscopic remission in ulcerative colitis

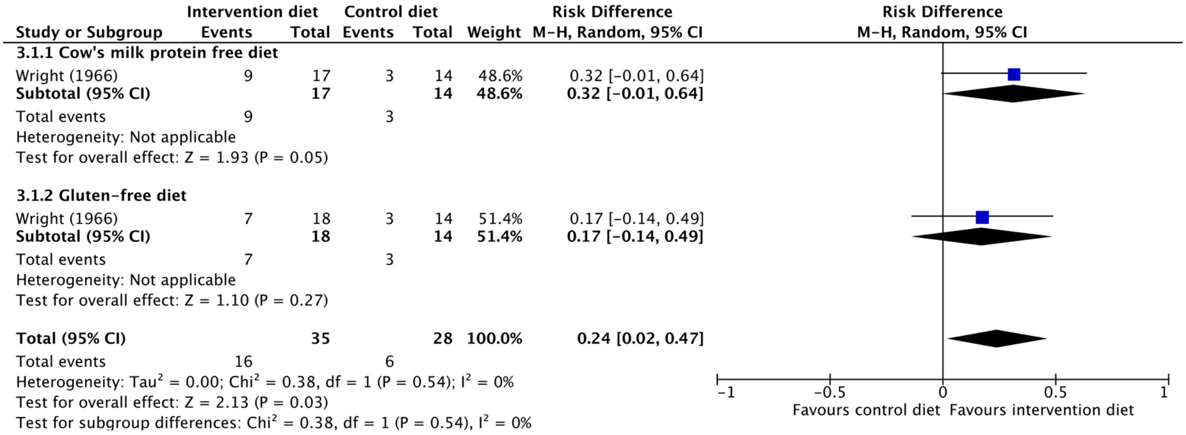

Figure S4. Induction of histologic remission in ulcerative colitis

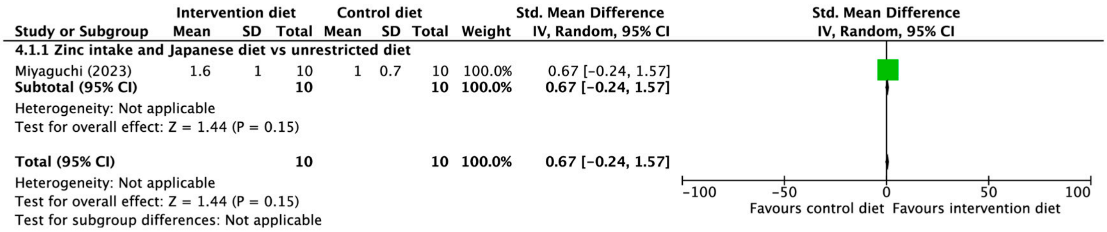

Figure S5. Histologic improvement in ulcerative colitis

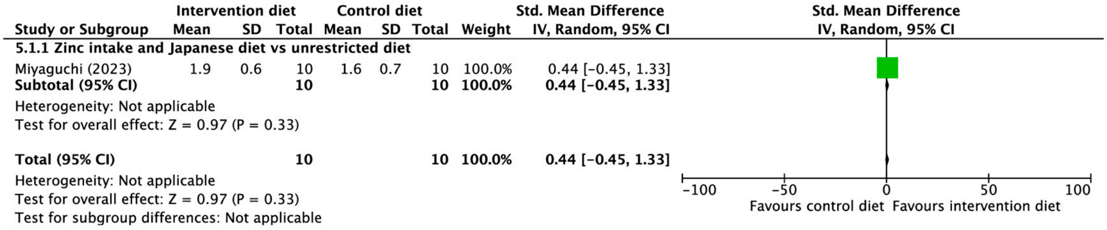

Figure S6. Endoscopic improvement in ulcerative colitis.
